# Surface-Enhanced Raman Spectroscopy Method for Sensitive Detection of Antibiotic Residues by Layer-Optimized Multilayer Nanoparticle Film Structures

**DOI:** 10.3390/nano16181184

**Published:** 2026-09-19

**Authors:** Hongyan Wang, Jialing Wei, Yu Hu, Cong Wang, Miao Qin

**Affiliations:** Key Laboratory of Spin Electron and Nanomaterials, Anhui Higher Education Institutes, School of New Energy Materials and Chemical Engineering, Suzhou University, Suzhou 234000, China

**Keywords:** surface-enhanced Raman scattering (SERS), hot spots, multilayer nanoparticle films, layer-dependent enhancement, antibiotic residues

## Abstract

Surface-enhanced Raman scattering (SERS) has been widely applied to food safety screening owing to its high sensitivity and fingerprint recognition. However, SERS faces challenges in practical applications related to the precise control of the number of hot spots and the determination of how many of them actually contribute to the collected signal. In this study, silver nanoparticles (AgNPs) were used to construct a series of layer-tunable AgNP film structures by assembling one to five layers of AgNP thin films using a liquid–liquid interface self-assembly method to obtain a large number of vertically coupled nanogap structures. The relationship between the stacking number and the effective enhancement was evaluated using crystal violet as a probe molecule. The results showed that the SERS intensity increased from one to three layers and then declined, mainly originating from the competition between the vertical plasmon coupling and the finite optical penetration depth; the point-to-point relative standard deviation of the crystal violet signal at 1617 cm^−1^, determined from 30 randomly selected positions, was lowest for the three-layer film (5.52%) and rose to 10.15% for the five-layer film. Finite element simulations and monolayer WS_2_ buried-probe measurements supported this result. Using the optimized three-layer AgNP film, four fluoroquinolone antibiotics were detected. The method was also applied to antibiotic residue screening in spiked chicken extracts. The layer-optimization approach may be adapted to other nanoparticle sizes and excitation wavelengths for broader SERS applications in environmental and food safety monitoring.

## 1. Introduction

Fluoroquinolones (FQs) are broad-spectrum synthetic antibiotics that are extensively used in the poultry industry for the prevention and treatment of bacterial infections [1]. Among these, ofloxacin (OFL), enrofloxacin (ENR), norfloxacin (NOR), and ciprofloxacin (CIP) are the most commonly administered in chicken farming owing to their high efficacy against both Gram-positive and Gram-negative bacteria [2]. However, the widespread and often indiscriminate use of these antibiotics has led to the accumulation of residues in animal-derived food products, which can cause allergic reactions, disrupt the intestinal microflora, and promote the emergence of antibiotic-resistant bacterial strains [3,4]. Therefore, regulatory authorities worldwide have established maximum residue limits (MRLs) for FQs in edible animal tissues; the European Union and China have both set an MRL of 100 μg/kg for the sum of FQ residues in poultry muscle [5]. Conventional analytical techniques for monitoring these residues include high-performance liquid chromatography (HPLC), liquid chromatography–tandem mass spectrometry (LC–MS/MS) [6,7]. Although HPLC and LC–MS/MS provide excellent sensitivity and accuracy, they require expensive instrumentation, lengthy sample pretreatment, and trained personnel, which limits their use in rapid on-site screening [8]. In contrast, SERS provides ultrahigh sensitivity together with molecular fingerprint recognition, requires minimal sample pretreatment, and can be implemented on portable instruments, and is therefore well suited to the rapid screening of antibiotic residues in poultry products [9,10]. In particular, the fingerprint specificity of SERS offers a route to discriminating the structurally similar FQ congeners that are difficult to resolve by immunoassay.

Since the initial recognition that the SERS enhancement is dominated by nanoscale gaps between adjacent metal structures [11,12], the origin of the Raman scattering enhancement in nanoparticle assemblies has been well explained, particularly the mechanism by which the local field is confined within interparticle gaps. By studying the signal distribution generated by nanoparticle arrays, researchers have shown that the enhancement factors span several orders of magnitude, and only a small fraction of the nanostructures produce enhancement factors large enough to dominate the collected signal [13]. Therefore, not all geometrically formed gaps are active SERS hot spots. Hot spots that are geometrically present but optically unreachable (usually due to variations in film thickness, stacking order, excitation wavelength, and collection depth) [14,15,16] lead to a wide distribution of effective enhancement factors. In previous studies, researchers have mainly focused on two aspects: the construction of hot spots and the improvement of the affinity between SERS substrates and target substances; in both cases the practical performance of a substrate is judged by the density and intensity of its hot spots together with its structural uniformity and signal reproducibility.

Prior work has shown that stacking nanoparticle films into multilayer structures creates a large number of vertically coupled small-gap hot spots [14,16,17]. A gap contributes to the collected signal only when two conditions hold: the excitation light must reach it (optical accessibility), and the analyte must reach it (molecular accessibility). Simulations and layer-resolved measurements of multilayer substrates indicate that the local field in the deeper gaps weakens progressively as the stack grows, so the enhancement cannot keep increasing with the layer number [15,16]. The second condition motivated the concept of interlayer small-gap structures that actively capture target molecules by nanoscale capillary action [18], and the electromagnetic contribution of a single interlayer gap was subsequently quantified by inserting a two-dimensional atomic crystal between two AgNP films [19]. Building on this, Wen et al. systematically examined Ag nanoparticle multilayers assembled at a liquid–liquid interface and showed, by finite element simulation together with SERS measurements, that the local field does not grow without bound with the layer number and that an optimum is reached within the first few layers [14,15]. Although the existence of such an optimum is therefore established, it has so far been inferred from simulations and from the overall signal collected at the film surface, and the excitation actually reaching a given depth inside the stack has not been measured directly; consequently, the physical origin of the optimum, and the depth at which the buried gaps cease to contribute, remain open. Multilayer nanoparticle films assembled at liquid–liquid interfaces, such as those of Au and Ag, have well-defined interparticle spacings [20] and a layer number that can be tuned one film at a time [21], which allows the hot spot population to be increased in controlled increments, thus allowing the enhancement to be examined as an explicit function of the stacking number. According to the electromagnetic enhancement mechanism of SERS, hot spots appear near sharp protrusions and form small gaps (nanometers or smaller) between metal nanostructures such as nanoparticles and nanofilms [22]. The gaps between the vertical metal nanostructures can achieve plasma multiple coupling, which helps generate greatly enhanced local electromagnetic fields [17]. However, the incident light is progressively depleted by absorption and scattering as it propagates through successive particle layers, so that the deeper gaps receive a diminished excitation. Therefore, it is feasible to construct a graded series of multilayer films and to combine theoretical simulation with a depth-resolved measurement to identify the stacking number at which the effective enhancement is maximized. Liu et al. showed that multilayer nanoparticle assemblies can excite bulk plasmon polaritons that redistribute the field across the stack, indicating that the field experienced by each layer depends on its depth within the assembly [23]. Chen et al. embedded a single layer of tungsten disulfide (WS_2_) as a Raman probe within a plasmonic nanocavity and measured the longitudinal plasmon field distribution through quantitative SERS intensity measurements of WS_2_ positioned at different depths [24].

In this study, we constructed one- to five-layer silver nanoparticle (AgNP) films using the liquid–liquid interface assembly method to obtain a graded series of vertically coupled gap structures. Throughout this work the layer number is a nominal layer number (NL), defined as the number of interfacial transfer cycles applied to the substrate. The relationship between the stacking number and the effective SERS enhancement was investigated using crystal violet (CV) as a probe molecule, and the finite element method was used to simulate the local electromagnetic field distribution of each structure. The simulations suggested that the non-monotonic dependence of the SERS intensity on the stacking number was mainly due to the finite penetration depth of the excitation laser, which corresponds to approximately three particle layers, and this was further verified experimentally by placing a single layer of WS_2_ beneath the AgNP film stack as a two-dimensional atomic probe. The optimized three-layer AgNP system was further used to detect four structurally related fluoroquinolones in standard solutions and in spiked chicken extracts. These results clarify how the stacking number governs the effective enhancement in multilayer gap systems and suggest a practical route for SERS-based food safety screening.

## 2. Materials and Methods

### 2.1. Materials and Reagents

Silver nitrate (AgNO_3_, 99.8%) and trisodium citrate dihydrate (C_6_H_5_Na_3_O_7_·2H_2_O, 99.0%) were used as the precursors for the synthesis of the silver nanoparticles. Cyclohexane and ethanol (AR) were employed as the organic solvents for the liquid–liquid interface assembly process. Crystal violet (C_25_H_30_ClN_3_, CV), used as the probe molecule for the SERS performance evaluation, was obtained from Sigma-Aldrich (Shanghai, China). Ofloxacin (C_18_H_20_FN_3_O_4_, OFL), enrofloxacin (C_19_H_22_FN_3_O_3_, ENR), norfloxacin (C_16_H_18_FN_3_O_3_, NOR), and ciprofloxacin (C_17_H_18_FN_3_O_3_, CIP), together with ethyl acetate and anhydrous sodium sulfate (Na_2_SO_4_), were purchased from Shanghai Aladdin Reagent Co., Ltd. (Shanghai, China). Ethyl acetate was used both as the extraction solvent for the chicken tissue and as the solvent for the antibiotic standard solutions, and anhydrous sodium sulfate was used to dry the organic extracts. WS_2_ monolayer continuous films were purchased from Six Carbon Technology (Shenzhen, China). All the chemicals used were of analytical grade and required no further purification. Ultrapure water (18.2 MΩ·cm) purified by a Millipore Milli-Q gradient system was used throughout all aqueous solution preparations and washing steps. Silicon wafers, used as the supporting substrates for the AgNP films, were cut into 0.5 × 0.5 cm pieces and rigorously cleaned with a mixture of ethanol and acetone prior to use.

### 2.2. Instrumentation and Operation

SERS and Raman measurements: All Raman and SERS spectra were recorded on a HORIBA Jobin Yvon XploRA PLUS confocal Raman microscope (Paris, France) equipped with a 633 nm excitation laser, a 600 lines mm^−1^ diffraction grating (1800 lines mm^−1^ for the WS_2_ spectra) and a thermoelectrically cooled Syncerity CCD detector (1024 × 256 pixels). A ×50 objective with a numerical aperture of 0.5 was used for all measurements. A built-in neutral-density filter reduced the laser output to 25% of its nominal power, which corresponds to a nominal 8.75 mW at the laser output. The power at the sample was lower because of losses in the optical path and was not measured.

Each spectrum was recorded as a single acquisition. The acquisition time was 1 s for the SERS spectra of CV, 3 s for the SERS spectra of the fluoroquinolones, and 10 s for the conventional Raman spectra of the solid fluoroquinolones and of the CV reference sample described below. For each SERS measurement, 10 µL of the analyte solution at the required concentration was dropped onto the prepared substrate and allowed to dry naturally before acquisition. Measurement positions were chosen at random within the central region of the substrate. The solid-state Raman spectra of the four fluoroquinolones were recorded from the powders, and each spectrum shown is the average of two acquisitions. As a reference without a SERS substrate, the conventional Raman spectrum of a 0.1 M CV solution on a bare silicon wafer was recorded with the same objective and laser setting, and the spectrum shown is the average of three acquisitions. All SERS spectra were baseline-corrected using LabSpec V5 software.

Scanning electron microscopy (SEM): SEM images were obtained using an Auriga focused ion beam scanning electron microscope (FIB-SEM) (Oberkochen, Germany), operating at an accelerating voltage of 15 kV.

Transmission electron microscopy (TEM): The AgNP colloid was diluted with anhydrous ethanol to a suitable concentration and sonicated for 5 min to disperse the particles. A 5 µL drop of the diluted colloid was placed on a carbon-supported copper grid for 1 min. The excess liquid was then removed from the edge of the grid with lint-free filter paper, and the grid was dried in air at room temperature. TEM images were recorded on a TF20 transmission electron microscope (Hillsboro, OR, USA) at an accelerating voltage of 200 kV, and the particle diameters were measured from the images with Nano Measurer 1.2.

Ultraviolet–visible (UV–Vis) spectroscopy: UV–Vis absorption spectra of the colloidal AgNPs were recorded on a Hitachi UH5700 spectrophotometer (Tokyo, Japan) fitted with a photomultiplier tube detector, to monitor their localized surface plasmon resonance. Spectra were recorded in transmission mode over 300–800 nm with a 1 nm sampling interval, using matched 1 cm quartz cuvettes with ultrapure water as the reference. Before measurement the colloid was diluted with ultrapure water at a colloid-to-water volume ratio of 1:3.

Electromagnetic field enhancement simulation software: COMSOL Multiphysics 6.4 software (Wave Optics Module) was used to simulate the local electromagnetic field distribution. The diameter of the AgNPs and the gap between the nanoparticles were set to 60 and 3 nm, respectively, with the 60 nm value close to the mean diameter of 64.4 nm measured by TEM (Section 3.1). Models representing one to five layers were built in a hexagonal close-packed arrangement, a perfectly matched layer was applied to the outer boundaries, and the incident wavelength was 633 nm, incident along the *Z*-axis.

### 2.3. Preparation of Citrate Silver Nanoparticles

The AgNPs with an average diameter of approximately 64 nm (Section 3.1) were synthesized via the classic citrate reduction method reported by Lee and Meisel [25]. A specific volume of AgNO_3_ aqueous solution (1 mM) was heated to a vigorous boil under continuous magnetic stirring in a round-bottom flask equipped with a reflux condenser. Upon boiling, a calculated amount of trisodium citrate solution (1 wt%) was rapidly injected into the reaction system. The solution was kept boiling for approximately 1 h, during which the color gradually transitioned from colorless to gray, and finally to a turbid yellow-green, indicating the formation of large-sized AgNPs. The resulting colloidal suspension was cooled to room temperature and stored in the dark at 4 °C for further assembly.

### 2.4. Assembly of Silver Nanoparticles at the Liquid–Liquid Interface

The self-assembly of AgNPs at the cyclohexane/water liquid–liquid interface was prepared according to the literature [26]. A portion of the as-synthesized AgNP colloid was concentrated by centrifugation to remove excess citrate and redispersed in water. Next, cyclohexane was added above the water layer, generating an immiscible cyclohexane/water liquid–liquid interface. Subsequently, ethanol was added dropwise into the interface under vigorous vibration; the Marangoni effect induced by the rapid diffusion of ethanol drove the AgNPs to be trapped at the oil–water interface, gradually forming a lustrous, mirror-like nanoparticle film at the interface. The self-assembled monolayer film was then transferred to a silicon wafer by carefully lifting the substrate from the bottom. After one layer of the AgNP film had dried, the film-picking process was repeated to produce AgNP thin films with two to five layers. Each film-picking cycle is counted as one nominal layer.

### 2.5. Construction of the Buried WS_2_ Depth-Probing Structure

Transfer of monolayer WS_2_: A high-precision two-dimensional material transfer platform was used for the alignment of WS_2_. The monolayer WS_2_/PDMS was placed on the opening fixture plate of the transfer platform for visualization of the precise transfer. The sample was heated to 90 °C, and the PDMS released viscosity to detach WS_2_ and transfer it to the target substrate.

Construction of the WS_2_/AgNP_n_ structure: According to the method described in the previous step, monolayer WS_2_ was transferred onto a bare silicon wafer. AgNP films with one to five layers were then sequentially deposited on top of the WS_2_ monolayer according to the method described in Section 2.4, so that the WS_2_ remained at a fixed depth at the base of the stack in every structure.

### 2.6. Pretreatment of Chicken Samples

Fresh chicken breast samples were purchased from a local supermarket. 5.0 g of homogenized chicken tissue was accurately weighed and placed into a 50 mL polypropylene centrifuge tube. For the spiked samples, an appropriate volume of the fluoroquinolone standard solution was added to the homogenized tissue to give the required spiked concentration, and the mixture was allowed to equilibrate at room temperature before extraction. Subsequently, 20 mL of ethyl acetate was added as the extraction solvent. The mixture was vortexed vigorously for 3 min and then subjected to ultrasonic extraction for 15 min at room temperature. After centrifugation at 8000 rpm for 10 min, the upper organic phase was collected and transferred to a clean tube. The extraction procedure was repeated once on the residual tissue with a further 20 mL of ethyl acetate, and the two organic phases were combined. The combined ethyl acetate extract was dried over anhydrous sodium sulfate to remove the residual water carried over from the tissue, and the desiccant was subsequently removed by filtration. The dried extract was evaporated to near dryness under a gentle nitrogen stream at 40 °C, reconstituted in 1 mL of ethyl acetate, filtered through a 0.22 µm syringe filter, and stored at 4 °C until SERS analysis. The antibiotic standard solutions were prepared in the same solvent, so that the standard solutions and the sample extracts were measured in a matched ethyl acetate system.

## 3. Results and Discussion

### 3.1. Characterization of Silver Nanoparticles

Figure S1a shows the UV–Vis absorption spectrum of the as-synthesized AgNP colloid. A narrow absorption band centered at approximately 418 nm confirms the presence of monodispersed, quasi-spherical silver nanoparticles without significant aggregation. The narrow bandwidth of this resonance indicates a sharp size distribution, which is a prerequisite for assembling films with uniform interparticle gaps. In addition, the surface of the AgNPs was quasi-spherical and uniform, as shown in Figure S1b. The particle size was measured on TEM images (Figure S2a). For 100 particles, the mean diameter was 64.4 ± 7.8 nm (mean ± SD; Figure S2b), a coefficient of variation of about 12%. Rod-shaped particles, seen occasionally in SEM images of the assembled films, were excluded from the size analysis. This diameter is consistent with the localized surface plasmon resonance peak position reported in the literature [27]. A particle size of this order is well matched to the 633 nm excitation used in this work because the dipolar resonance of the assembled film is thereby positioned to couple efficiently with the incident field.

**Scheme 1:**
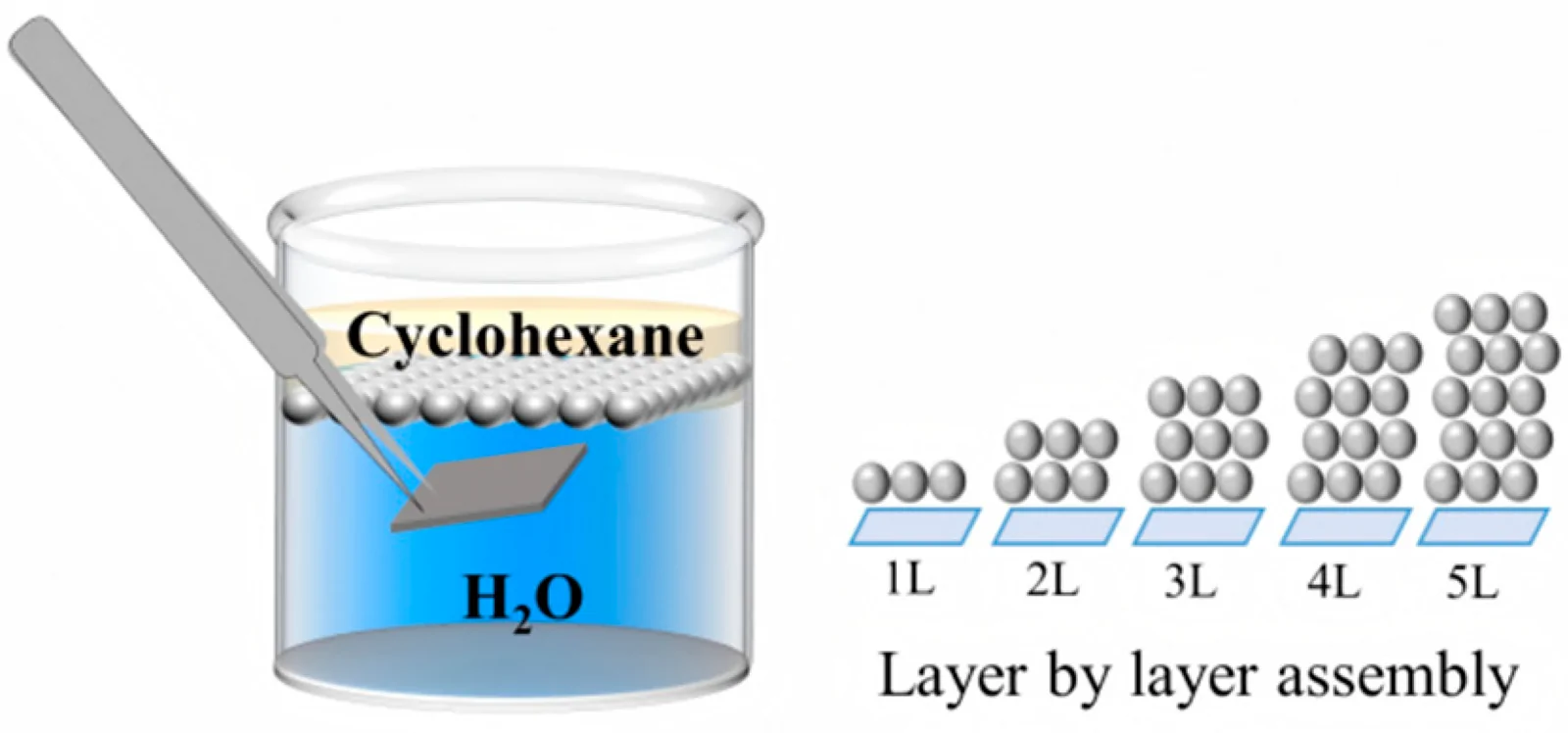
Schematic illustration of the self-assembly of AgNPs at the cyclohexane/water interface, the transfer of the interfacial film onto a silicon wafer, and the layer-by-layer construction of one- to five-layer (1–5 L) AgNP films by repeated transfer cycles.

After determining that the AgNPs were monodispersed and of a suitable size, we characterized their behavior at the liquid–liquid interface. When ethanol was introduced into the cyclohexane/water interface, the AgNPs migrated to the interface and formed a continuous, lustrous, mirror-like film within seconds (Scheme 1). The interfacial film could be lifted onto a silicon wafer without visible cracking, and the transferred film retained its metallic luster after drying (Scheme 1), indicating that a single transfer cycle deposits one continuous nanoparticle layer. Each transferred monolayer thus served as the building block for the multilayer structures described below.

### 3.2. Construction and Characterization of Multilayer AgNP Film Structures

**Figure 1:**
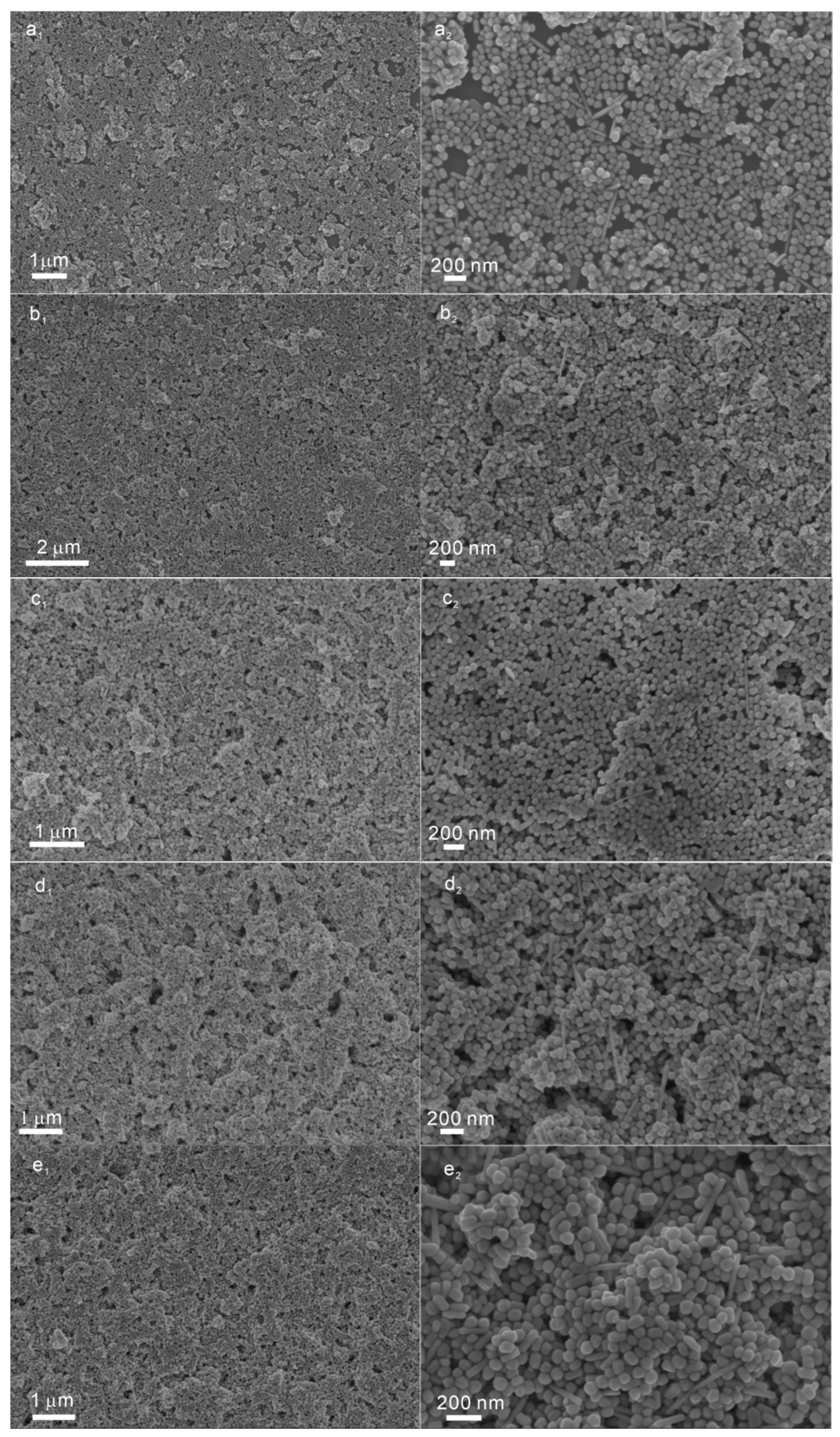
SEM images of the (**a_1_**,**a_2_**) one-layer, (**b_1_**,**b_2_**) two-layer, (**c_1_**,**c_2_**) three-layer, (**d_1_**,**d_2_**) four-layer, and (**e_1_**,**e_2_**) five-layer AgNP films. Panels (**a_1_**–**e_1_**) were recorded at low magnification (scale bars: 1 µm, except (**b_1_**): 2 µm) and panels (**a_2_**–**e_2_**) at high magnification (scale bars: 200 nm).

A series of one- to five-layer AgNP film structures was constructed using a liquid–liquid self-assembly method. To further understand the generation of a large number of vertically coupled gap structures between the stacked AgNP films, we performed SEM characterization of the films with different layer numbers (Figure 1). A large quantity of approximately 3 nm gaps formed between the AgNPs, providing a structural basis for the subsequent generation of interlayer hot spots. Figure 1(a_1_,a_2_) shows the morphology of the monolayer film, where the AgNPs are arranged in a relatively dense two-dimensional planar structure. Although the interfacial tension drives the particles to pack closely, small interparticle voids and sub-monolayer defects are inevitable due to the steric hindrance of the capping agents and the rapid solvent evaporation process. Upon the deposition of a second layer (Figure 1(b_1_,b_2_)), surface coverage improves noticeably; the second-layer nanoparticles fill the interstitial sites and voids of the first layer, producing a more compact arrangement with incipient vertical coupling. For the three-layer film (Figure 1(c_1_,c_2_)), the surface is densely packed and rough, with AgNPs stacked extensively in the vertical direction; the transition from a two-dimensional planar array to a three-dimensional stacked architecture is essentially complete at this stage. In the four-layer (Figure 1(d_1_,d_2_)) and five-layer (Figure 1(e_1_,e_2_)) films, the accumulation of nanoparticles becomes even more pronounced, leading to a thicker film with increased surface roughness, although the surface morphology no longer changes qualitatively. The images of the thicker films also show darker regions. At these magnifications we interpret them as voids between the topmost particles, but top-view SEM images cannot distinguish such voids from local cracking or delamination, and the present films were not imaged in cross-section.

Because the multilayer films are the analytical surface in this work, keeping the stack intact during measurement matters as much as its enhancement. Self-assembled nanoparticle substrates are usually judged by their point-to-point uniformity and batch-to-batch reproducibility [28]. The liquid–liquid interfacial transfer used in this work has previously been applied to two-layer AgNP films, for which cross-sectional FIB-TEM imaging showed a continuous stack separated by sub-3 nm interlayer gaps [18]. Here, the SEM images in Figure 1 show the coverage and morphology at each nominal layer number, and three further observations indicate that the films stayed continuous on the scale probed by the laser spot throughout the measurements. First, the layer dependence of the CV signal was reproduced at two analyte concentrations an order of magnitude apart. Second, the point-to-point variation in the CV signal on the four- and five-layer films was of the same order as on the thinner films (Section 3.3), which would not be expected if the thicker stacks had broken up extensively. Third, the buried WS_2_ signal decreased with the number of covering layers. The three- to five-layer films were not examined by cross-sectional microscopy or atomic force microscopy in this work, however, so their internal stacking is inferred rather than imaged, and the layer number is reported as a nominal value.

### 3.3. Layer-Dependent SERS Enhancement Effect of the Multilayer AgNP System

**Figure 2:**
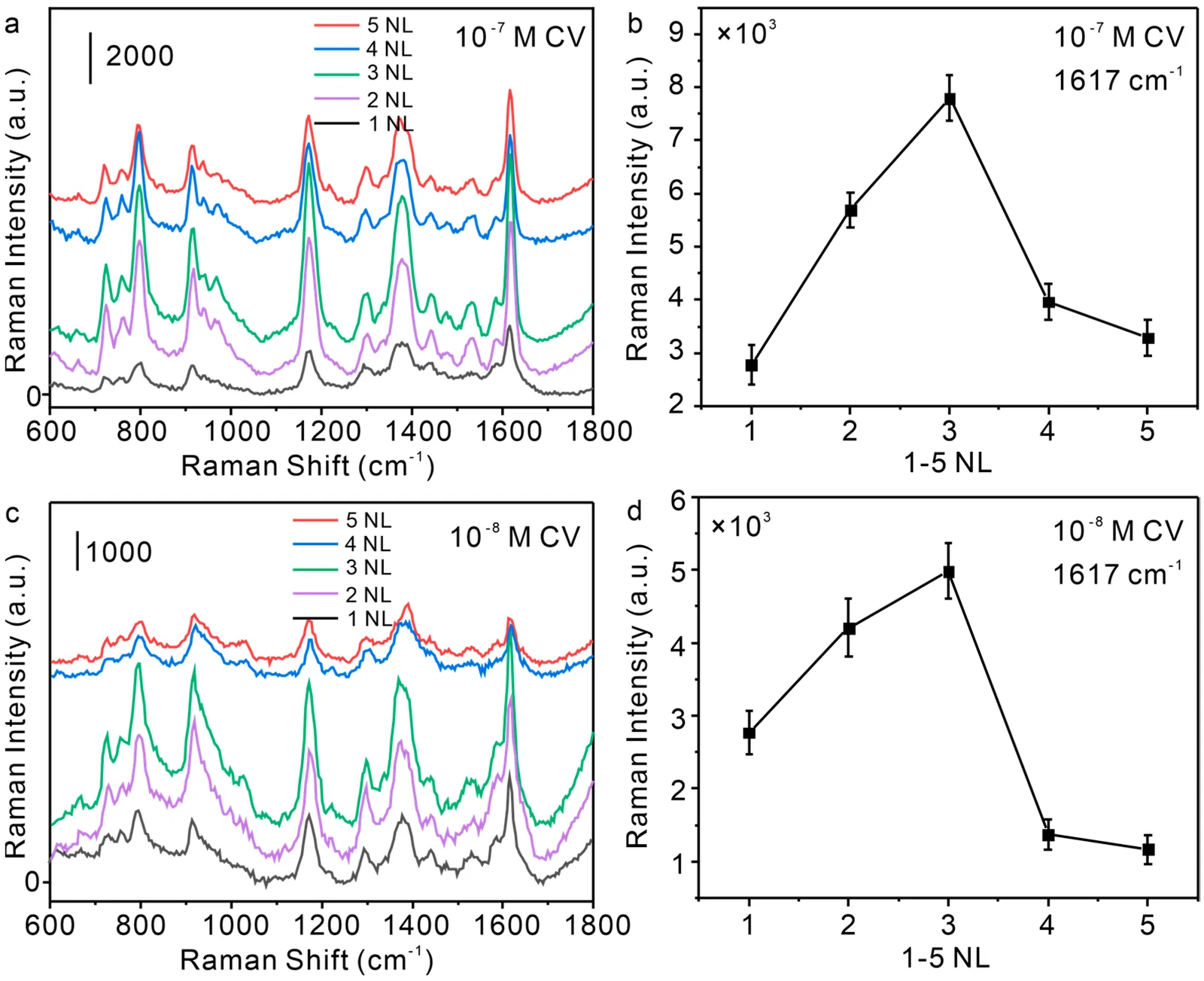
(**a**) SERS spectra of 10^−7^ M CV on the one– to five–layer AgNP films. (**b**) Corresponding peak intensity at 1617 cm^−1^ as a function of nominal layer number. (**c**) SERS spectra of 10^−8^ M CV on the one– to five–layer AgNP films. (**d**) Corresponding peak intensity at 1617 cm^−1^ as a function of layer number. The excitation wavelength was 633 nm, and the accumulated time was 1 s.

We next investigated the layer dependence of the electromagnetic field enhancement by measuring the SERS intensity of CV, because the Raman signal is directly related to surface plasmon coupling between adjacent nanounits [29]. The stacked AgNP films, containing both intralayer and interlayer gaps, are well suited for studying the relationship between stacking number and effective enhancement [30,31]. The strong coupling between the vertically distributed AgNPs was expected to cause significant electromagnetic hot spots at the interlayer gaps, enhancing the Raman signal of the adsorbed CV. The SERS spectra of 10^−7^ M CV on the one- to five-layer AgNP films (Figure 2a) and of 10^−8^ M CV on the same series (Figure 2c) were collected. Three strong characteristic peaks associated with CV were observed, which were the ring skeletal, C–H bending, and C–C ring stretching peaks at 920, 1172, and 1617 cm^−1^, respectively. After the deposition of the third AgNP layer, the SERS intensity of CV significantly increased compared to that on the monolayer film, indicating that the surface plasmons of the metal interlayer nanostructure were strongly excited.

We further investigated the enhancement by dividing the SERS intensity of CV at 1617 cm^−1^ as a function of layer number. As shown in Figure 2b,d, the enhancement ratio increased progressively from the one-layer to the three-layer film at both concentrations, and the intensity variation trends measured at 10^−7^ and 10^−8^ M were mutually consistent, confirming that the observed layer dependence is not an artifact of the analyte concentration. When the stacking number exceeded three, the enhancement ratio did not continue to increase but instead declined and then stabilized at a lower level. To quantify the point-to-point uniformity of each film, the RSD of the CV band at 1617 cm^−1^ was calculated from the 30 spectra recorded at 10^−7^ M on each film (Figure S4). The RSD was 13.53%, 5.92%, 5.52%, 8.60% and 10.15% for the one- to five-layer films, respectively. The monolayer varied most, consistent with the interparticle voids and sub-monolayer defects visible in Figure 1a. The second and third layers fill these voids, and the three-layer film gave the lowest RSD. In the four- and five-layer films the particles were transferred onto an increasingly rough surface, which fits the renewed rise in signal fluctuation. Of the five films, the three-layer film therefore has both the highest CV intensity and the lowest point-to-point variation. As the three-layer silver nanoparticle film exhibited the highest CV detection intensity for samples of the same concentration, a CV sensitivity test was conducted on the three-layer AgNP films, as shown in Figure S5. The minimum identifiable analysis signal Sm should be at least equal to the sum of the mean S of blank signal plus 3 times the standard deviation (SD) of blank signal [32]: *S*_m_ = *S* + 3SD, the experimental method for measuring Sm is to obtain S and SD through statistical treatment through 20–30 blank measurements within a certain period of time. According to this experiment, the blank Ag nanoparticle film substrate was collected for 30 times (920 cm^−1^), as shown in the Figure S6. After data statistical processing, S_m_ = 157 + 3 × 36 = 265. For our system, as shown in the Figure S7, when the detection concentration is 10^−10^ M, the signal intensity of CV at 920 cm^−1^ is 762 (a.u.), which is within the recognizable range. Because the geometric hot spot density continues to grow with every added layer, the decline in enhancement beyond three layers cannot be explained by the structural evolution alone; we therefore examined the depth over which the stacked hot spots remain optically accessible, using theoretical simulation and a depth-resolved measurement in turn.

**Figure 3:**
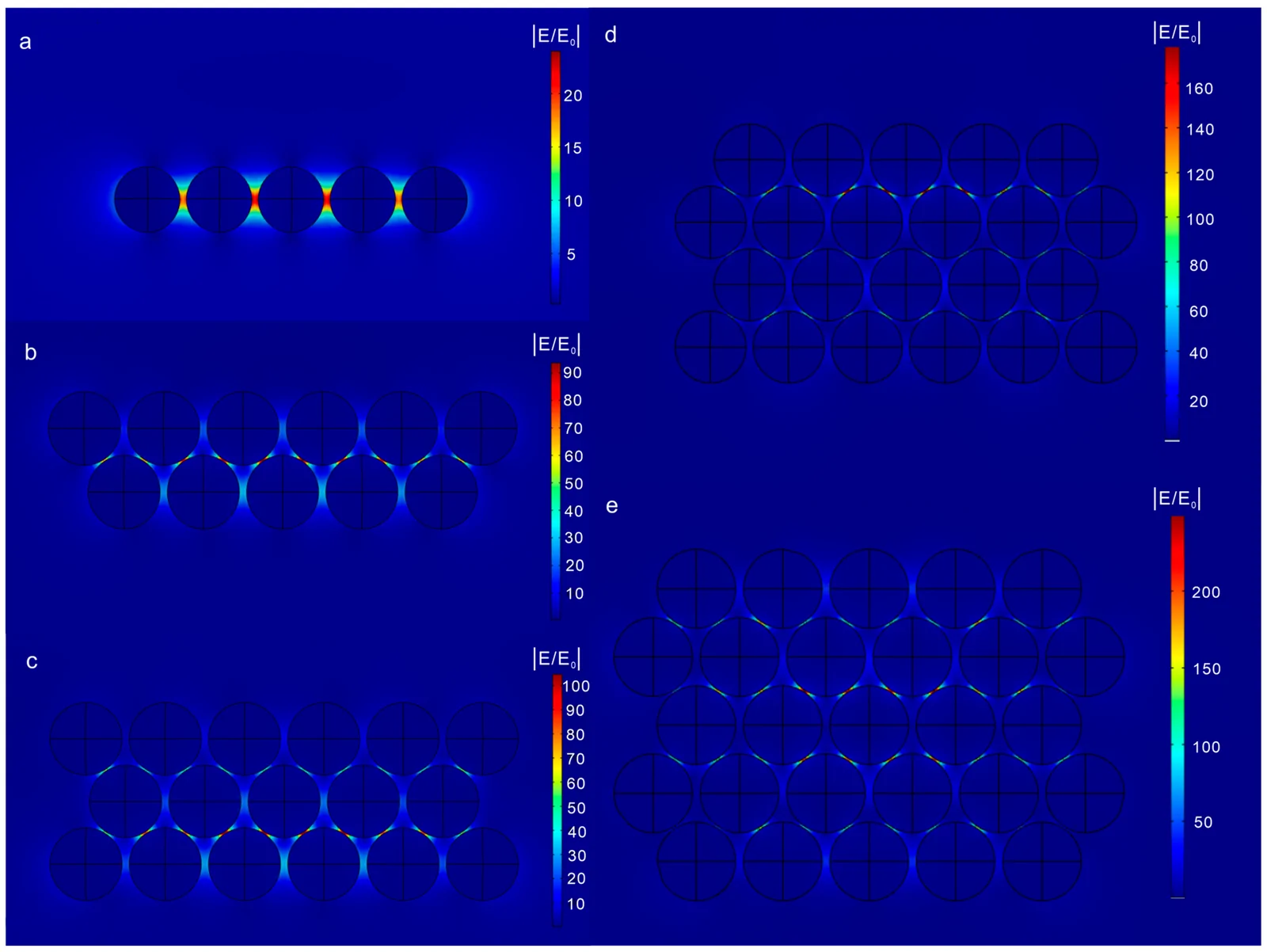
The cross-sectional near-field profiles of the multilayer AgNP structures at an excitation wavelength of 633 nm: (**a**) one–layer; (**b**) two–layer; (**c**) three–layer; (**d**) four–layer; (**e**) five–layer.

To better understand the nature of the layer-dependent electromagnetic field enhancement in the multilayer AgNP system, we conducted a theoretical simulation study of the system in which the optical interactions between AgNPs in the same layer and between AgNPs in different layers were fully considered. In our current structure, the coupling between particles (3 nm gap) is very close, such that both intralayer AgNP–AgNP and interlayer AgNP–AgNP couplings exist simultaneously. To investigate the plasmon coupling effect in the proposed structure, we performed numerical simulations based on the finite-element method [17]. As shown in Figure 3a–e, one to five AgNP arrays were stacked along the z-direction. The diameter of the AgNPs was set to 60 nm and the distance between adjacent AgNPs was 3 nm. In the simulation, the plane light wave was normally incident from the top in the z-direction. For the one- and two-layer structures, the hot spots were largely confined to the lateral interstices between adjacent nanoparticles within the plane (Figure 3a,b). For the three-layer structure, hot spots appeared in the interlayer gaps and the field extended into the second and third layers (Figure 3c); the maximum |E/E_0_| in this structure was approximately 103, corresponding to a maximum simulated value of |E/E_0_|^4^ in the hot spot of approximately 1.1 × 10^8^ [17]. This local, simulated value contains neither chemical nor resonance contributions and is not an experimental enhancement factor, which is why it is larger than the spatially averaged analytical enhancement estimated above. When the stacking number was increased to four and five layers, the distribution of the electric field became highly non-uniform along the vertical direction: although the maximum local field at the topmost surface was even higher than in the three-layer structure, the field in the bottom layers decayed markedly, appearing as dark regions in the simulation maps (Figure 3d,e). The peak local field therefore does not single out the three-layer structure. Instead, the simulations show that beyond three layers the additional field is confined to the upper part of the stack and the buried gaps are only weakly excited. This behavior reflects the skin effect and the limited optical penetration depth of the laser in metallic nanostructures; once the film thickness exceeds the effective penetration depth, the incident light is largely absorbed or scattered by the upper layers and cannot excite the plasmons of the underlying particles. A comparable behaviour has been exploited deliberately in porous plasmonic membranes, where the metal overlayer suppresses the penetration of the excitation laser into the material beneath it and thereby confines the Raman response to the topmost few nanometres [33]. Moreover, the Raman photons generated in the deeper layers are subject to reabsorption and scattering losses as they propagate back to the surface, so that this parasitic absorption further suppresses the contribution of the buried gaps to the collected signal. Hot spots buried deeper receive too little laser intensity to contribute meaningfully to the collected signal. The measured signal also depends on how much excitation reaches the buried gaps and how much of the Raman light generated there escapes the film, and the peak field value captures neither. We therefore measured the depth over which the stacked gaps contribute to the collected signal, using the buried WS_2_ probe described below.

**Figure 4:**
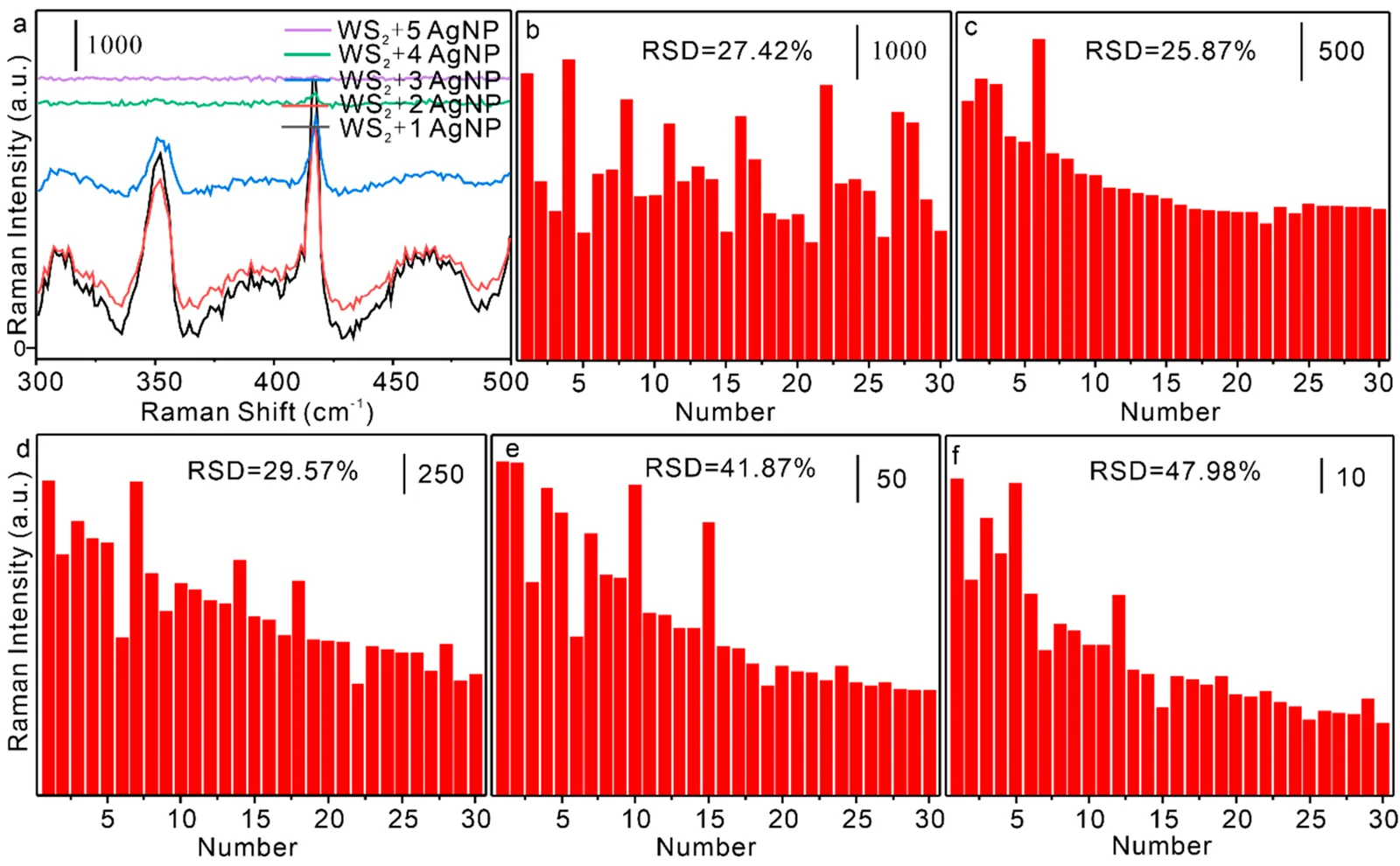
(**a**) Raman spectra of monolayer WS_2_ buried beneath one– to five–layer AgNP films. (**b**–**f**) RSD analysis of the buried WS_2_ signal collected from 30 randomly selected positions under the (**b**) one–layer, (**c**) two–layer, (**d**) three–layer, (**e**) four–layer, and (**f**) five–layer coverage. In (**a**), the tick labelled 0 marks the zero intensity of the lowest trace, and the vertical bar indicates the intensity scale. In (**b**–**f**), each bar is the intensity of the WS_2_ A_1g_ band at 419 cm^−1^ at one of the 30 positions; the ordinate of each panel starts at zero at the lower frame, the vertical bar indicates the intensity scale of that panel, and the RSD of the 30 values is given in each panel.

To experimentally verify the penetration depth predicted by the simulation, monolayer WS_2_ was placed beneath the AgNP stack as a fixed-depth Raman marker. Monolayer WS_2_ was chosen because it is chemically inert, sub-nanometer thick, and optically transparent in the visible range, so that its insertion does not perturb the stacking geometry of the AgNP film above it. The Raman spectrum of WS_2_ on a bare silicon wafer showed two characteristic shifts at 352 cm^−1^ and 419 cm^−1^, As shown in Figure S8, which were attributed to the in-plane E_2g_ and out-of-plane A_1g_ phonon vibration modes of monolayer WS_2_, respectively, consistent with the Raman peaks and layer thickness reported in the literature [24]. The spectra of the buried WS_2_ covered by one to five layers of AgNPs are shown in Figure 4a. The maximum Raman intensity was observed when the WS_2_ was covered by only a single layer of AgNPs, which can be attributed to the strong localized electromagnetic field generated at the AgNP–WS_2_ interface. As the number of AgNP layers increased to two and three, a progressive attenuation in the WS_2_ signal intensity was observed; when the overlayer thickness reached four and five layers, the characteristic peaks of WS_2_ almost completely vanished into the baseline noise. This monotonic decrease shows that the upper AgNP layers act as an optical shield, blocking both the incoming laser and the outgoing Raman photons. RSD analysis of the buried signal collected from 30 random positions (Figure 4b–f) showed that the RSD increased from 27.42% for the one-layer coverage to 47.98% for the five-layer coverage, which is a direct consequence of the decreasing signal-to-noise ratio caused by the blocked excitation light. For the four- and five-layer coverages the WS_2_ peak height is close to the noise level of the measurement, so the RSD values quoted for these two structures are dominated by noise and are reported only for completeness. Because the WS_2_ is held at a fixed depth in every structure, the decay of its signal reports the residual excitation reaching the base of the stack rather than any change in the marker itself. Through these theoretical and experimental approaches, it can be concluded that the incident light is largely depleted before reaching the fourth particle layer, and that the contribution of the gaps buried beyond the third layer is much smaller. The simulations predict stronger local fields in the four- and five-layer stacks (Figure 3d,e), but the buried WS_2_ probe shows that attenuation of the incident laser and of the outgoing Raman light limits how much these gaps add to the measured signal. For detection, the three-layer AgNP film therefore gives the best balance between the number of vertically coupled hot spots and how well they can be reached by the excitation light and read out, which agrees with the highest CV signal being measured on this film (Figure 2b,d).

### 3.4. Detection of Fluoroquinolone Standards by the Optimized Three-Layer AgNP Film

**Figure 5:**
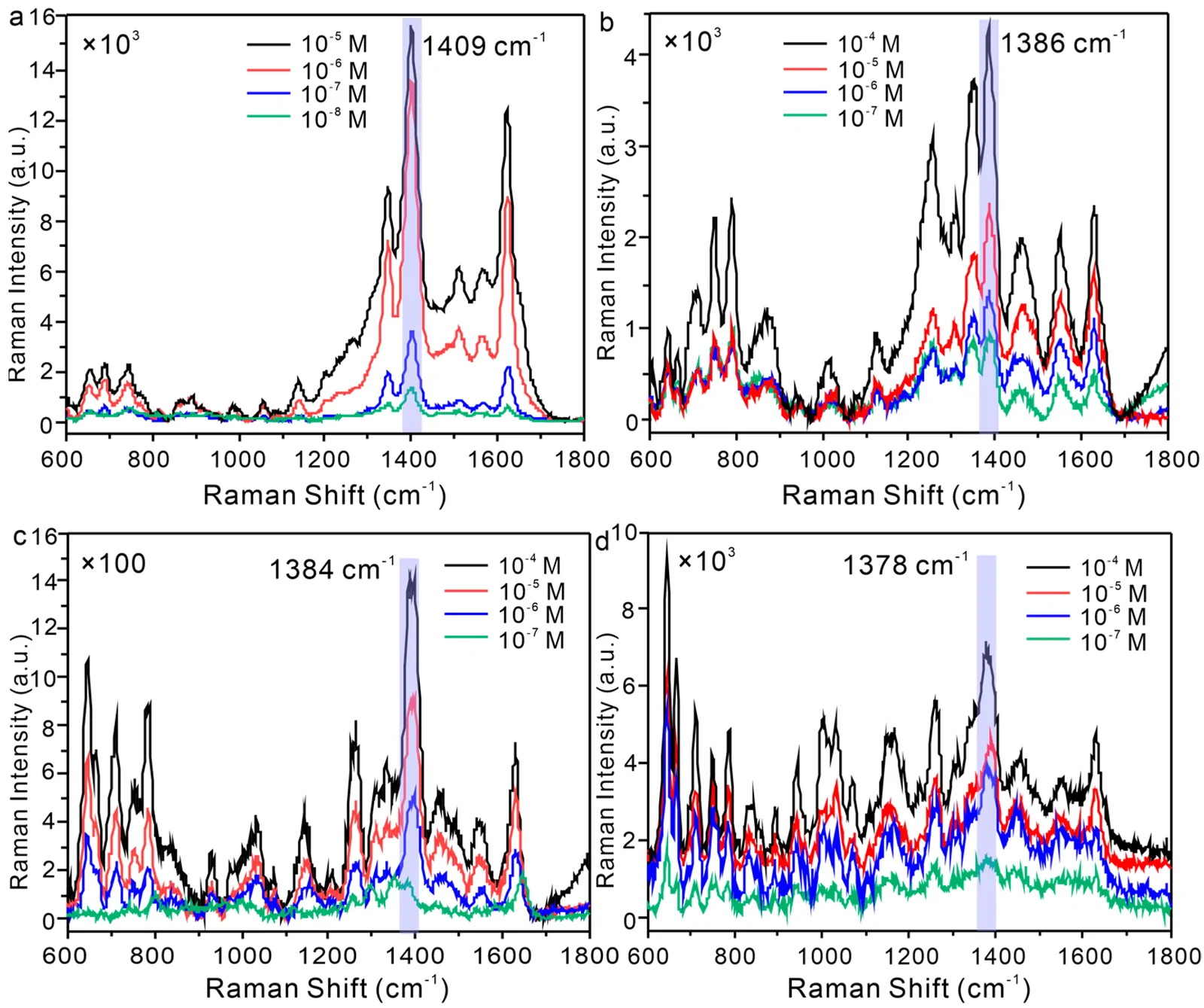
SERS detection of four fluoroquinolone antibiotics in the optimized three–layer AgNP film. (**a**) OFL solutions from 10^−5^–10^−8^ M. (**b**) ENR solutions from 10^−4^–10^−7^ M. (**c**) NOR solutions from 10^−4^–10^−7^ M. (**d**) CIP solutions from 10^−4^–10^−7^ M.

As described above, FQ residues in poultry products are widely distributed, closely related in structure, and regulated at trace levels, which makes their rapid discrimination a demanding test of any SERS substrate. The optimized three-layer AgNP film was used to detect four of these compounds. The solid-state Raman spectra of the four antibiotics were collected to obtain their intrinsic molecular fingerprint information (Figure S9). The four compounds share a common quinolone skeleton but differ in their piperazine substituents and side groups, so that their Raman spectra contain both a set of common skeletal bands and a set of substituent-sensitive bands that permit discrimination. The main solid-state bands of OFL, ENR, NOR and CIP, the SERS bands subsequently observed on the three-layer film (Figure 5), and their tentative assignments according to refs [34,35,36,37,38,39] are summarized in Table S1, and the solid-state spectra were used as the reference for identifying the SERS bands. One band was selected for each compound to construct the intensity–concentration relationship, namely, 1409 cm^−1^ for OFL, 1386 cm^−1^ for ENR, 1384 cm^−1^ for NOR and 1378 cm^−1^ for CIP (shown in bold in Table S1). These bands were chosen because each shows a clear concentration-dependent response (Figure S10) and because the four bands are distributed across the 1370–1410 cm^−1^ region and can be resolved from one another, which improves the reliability of single-component identification.

Solid-state bands were read from the solid-state Raman spectra (Figure S9) and SERS bands from the spectra of the ethyl acetate standard solutions on the three-layer AgNP film (Figure 5); solid-state and SERS bands lying within 10 cm^−1^ of each other are placed on the same row. A literature assignment is given for a row only when its solid-state band lies within 10 cm^−1^ of a powder band, or its SERS band within 10 cm^−1^ of a SERS band, reported for the same compound in the cited work; “—” indicates that no such assignment was found. Where two cited works assign the same band differently, both assignments are given. Bands shown in bold were used to construct the intensity–concentration relationships (Figure S10). No ofloxacin assignment lying within 10 cm^−1^ of these bands was found in the ofloxacin studies cited [38,39]; these bands are assigned by analogy with the corresponding bands of structurally related fluoroquinolones reported in the cited works.

The optimized three-layer AgNP film was then used to detect the four fluoroquinolones in ethyl acetate standard solutions, as shown in Figure 5. OFL is a fluoroquinolone widely used for the treatment of respiratory and intestinal infections in poultry. As shown in Figure 5a, OFL standard solutions from 10^−5^ to 10^−8^ M exhibit distinguishable SERS signals, with the main bands located at 751, 1345, 1409, and 1621 cm^−1^ (Table S1), with OFL detectable down to 10^−8^ M. Among these bands, the peak at 1409 cm^−1^ shows a relatively strong intensity and a clear concentration-dependent response, and was therefore selected as the characteristic peak for constructing the peak intensity–concentration relationship. ENR is a fluoroquinolone developed specifically for veterinary use. As shown in Figure 5b, the SERS spectra recorded from 10^−4^ to 10^−7^ M exhibit characteristic peaks at 790, 1256, 1386, 1464, 1549, and 1630 cm^−1^ (Table S1), and the detection limit for ENR is 10^−7^ M. These peaks are generally consistent with the main vibrational features observed in the solid Raman spectrum, although slight shifts occur because molecular adsorption on the AgNP surface alters the local chemical environment and the SERS selection rules favour the vibrational modes oriented perpendicular to the metal surface; the band at 1386 cm^−1^ was selected for the quantitative analysis. NOR is one of the most frequently detected residues in poultry muscle. As Figure 5c shows, NOR was detected down to 10^−7^ M over the range from 10^−4^ to 10^−7^ M, and the band near 1384 cm^−1^ (Table S1), which is associated with the quinolone skeleton and carboxyl-related vibrations [34,36], was selected as the representative characteristic peak. CIP is the principal active metabolite of ENR and therefore frequently co-occurs with it in poultry tissue. In Figure 5d, the main SERS peaks appear at 1026, 1134, and 1378 cm^−1^ (Table S1) over the range from 10^−4^ to 10^−7^ M, with a detection limit of 10^−7^ M; compared with the solid Raman peak at 1384 cm^−1^, the SERS peak shifts slightly to 1378 cm^−1^, which can be attributed to the change in the local chemical environment after adsorption on the Ag surface, and this band was selected for the quantitative analysis.

To further evaluate the concentration-dependent response of the optimized substrate, the peak intensities of the four selected characteristic bands were plotted against the logarithm of the antibiotic concentration (Figure S10). The selected peaks at 1409 cm^−1^ for OFL, 1386 cm^−1^ for ENR, 1384 cm^−1^ for NOR, and 1378 cm^−1^ for CIP all exhibit clear positive correlations with the logarithmic concentration, and the signal intensity decreases monotonically as the analyte concentration decreases. The differences in SERS signal intensity among the four antibiotics may be related to their molecular structures, adsorption affinities, and orientations on the AgNP surface. Fluoroquinolones contain multiple functional groups, including carboxyl, carbonyl, fluorinated aromatic rings, and nitrogen-containing heterocycles, which can interact with the Ag surface to different extents; these interactions affect the distance and orientation between the molecular vibration units and the interlayer hot spots, thereby influencing the final SERS enhancement. The detection limits of OFL, ENR, NOR, and CIP were approximately 10^−8^ M, 10^−7^ M, 10^−7^ M, and 10^−7^ M, respectively.

The SERS spectra of the four antibiotics (Figure 5) differ from their solid-state Raman spectra (Figure S9) both in the positions of individual bands and in their relative intensities, and several mechanisms may contribute to these differences. First, the surface selection rules of SERS preferentially amplify vibrational modes whose polarizability derivative has a component normal to the metal surface, so that the adsorption orientation of the molecule determines which modes are enhanced most strongly and the relative intensities in the SERS spectrum need not follow those of the solid [22]. Second, a chemical (charge-transfer) contribution arising from the molecule–metal interaction can further redistribute the relative intensities of individual bands [40]. Third, adsorption on the silver surface perturbs the local chemical environment of the molecule and produces small frequency shifts; the quantitative bands of NOR and CIP illustrate this, appearing at 1384 and 1378 cm^−1^ in the SERS spectra compared with 1410 and 1384 cm^−1^ in the solid-state spectra. Fourth, the two measurements probe different states of the compound: the solid-state spectrum reflects molecules in a crystalline lattice, where packing and hydrogen bonding influence the vibrational frequencies, whereas the SERS spectrum reflects essentially isolated molecules adsorbed on the silver surface from dilute solution. These mechanisms are likely to act together, and the assignments in Table S1 are therefore given as tentative.

### 3.5. Detection of Fluoroquinolone Residues in Spiked Chicken Extracts

To evaluate the optimized three-layer AgNP film in a real sample matrix, chicken breast tissue was used as the sample, ethyl acetate was used to extract the antibiotic molecules from the spiked tissue, anhydrous sodium sulfate was used to dry the combined organic extracts, and SERS detection was performed directly on the blank extract to establish the influence of its intrinsic signals. As shown in Figure S11, the ethyl acetate blank measured on the same substrate shows weak bands, the most prominent near 935 and 1300 cm^−1^, whereas the blank chicken extract shows these features with markedly higher intensity, together with a higher and more structured background across the fingerprint region, which may arise from co-extracted lipids, amino acid-related residues and small metabolites [41]. The chicken matrix is therefore not spectroscopically silent; compared with the pure ethyl acetate system, the more complex background of the extract gives rise to partial peak overlap, baseline fluctuation, and overall signal attenuation, all of which must be taken into account when the spiked spectra are interpreted. Accordingly, the spiked spectra were interpreted by comparing the characteristic antibiotic peaks selected in Section 3.4 with the blank matrix background, rather than by reporting a statistical detection limit without blank-corrected calibration. This treatment is more appropriate for the feasibility evaluation and the rapid screening scenario targeted in this work.

**Figure 6:**
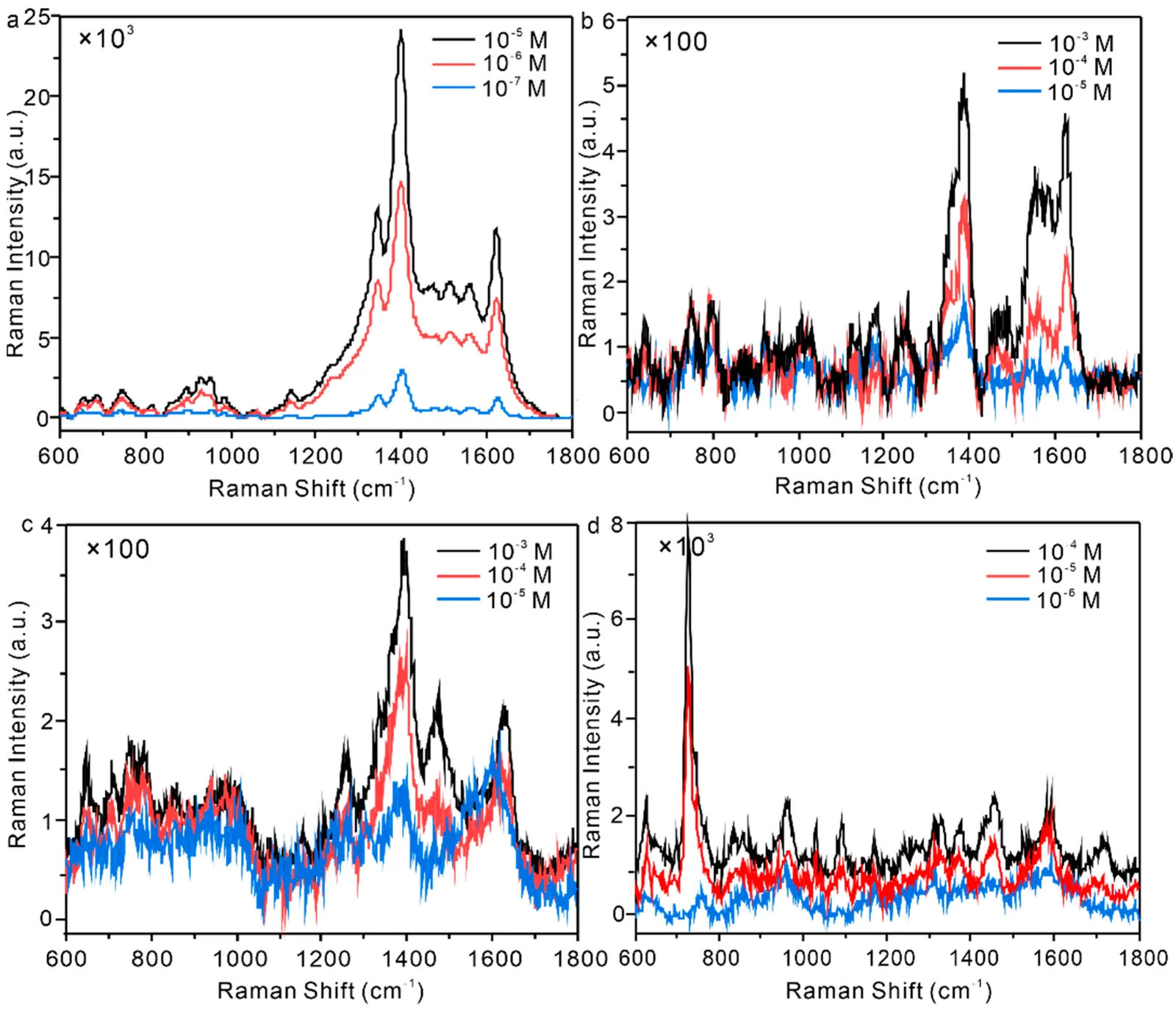
SERS spectra of four fluoroquinolone antibiotics in spiked chicken extracts: (**a**) OFL at 10^−5^–10^−7^ M; (**b**) ENR at 10^−3^–10^−5^ M; (**c**) NOR at 10^−3^–10^−5^ M; (**d**) CIP at 10^−4^–10^−6^ M.

The SERS spectra of the antibiotic-spiked chicken extracts are shown in Figure 6. For OFL (Figure 6a), the characteristic features remain distinguishable at spiked concentrations of 10^−5^, 10^−6^, and 10^−7^ M, especially around the selected peak at 1409 cm^−1^, and the signal intensity decreases as the concentration decreases, indicating a concentration-dependent response in the chicken matrix. Compared with the ethyl acetate standard solution, in which OFL could be identified at 10^−8^ M, the lowest identifiable concentration in the chicken extract shifts to 10^−7^ M; this decrease in sensitivity can be attributed to competitive adsorption of matrix molecules on the AgNP surface and partial interference from the endogenous signals. For ENR (Figure 6b) and NOR (Figure 6c), the spiked spectra recorded at 10^−3^, 10^−4^, and 10^−5^ M exhibit more pronounced background fluctuation, suggesting stronger matrix interference in the 1380–1390 cm^−1^ region where their selected peaks are located; nevertheless, the characteristic responses near 1386 and 1384 cm^−1^ can still be observed and decrease as the spiked concentration decreases, and the lowest identifiable concentration for both compounds is 10^−5^ M. The larger shift relative to the standard solution indicates that the chicken matrix suppresses the effective SERS response of ENR and NOR more strongly, possibly because of their weaker adsorption competitiveness against the co-extracted matrix components. In contrast, for CIP (Figure 6d), the characteristic peak near 1378 cm^−1^ remains visible at spiked concentrations down to 10^−6^ M, indicating relatively favourable detectability compared with ENR and NOR in the same matrix; this may be related to the adsorption configuration of CIP on the AgNP surface and to the fact that its selected band lies at the low-wavenumber edge of the interfering region.

Overall, the optimized three-layer AgNP film enables the detection of all four representative fluoroquinolones in spiked chicken extracts, with the lowest identifiable concentrations being 10^−7^ M for OFL, 10^−6^ M for CIP and 10^−5^ M for ENR and NOR. The experimental results showed that in a complex environment of chicken extracts, the prepared three-layer AgNP structure can be used to detect the antibiotic residue signal in real tissue, confirming the applicability of this substrate for SERS screening of antibiotic residues in complex matrices.

The present evaluation has several limitations. First, the analyte was deposited as a 10 µL droplet and left to dry, and the lateral distribution of the dried deposit was not mapped. Because the measurement positions were chosen at random within the central region of the substrate, an uneven distribution of the analyte towards the edge of the dried droplet cannot be excluded, and the RSD values reported here describe the point-to-point variation within that central region only. Second, all replicate measurements were made at different positions on a single substrate. They therefore characterize the within-substrate repeatability of the method, whereas the substrate-to-substrate and batch-to-batch reproducibility, an important criterion for practical SERS substrates [20,28,32], was not evaluated in this work. However, challenges remain in expanding this method to other areas of food safety monitoring. These include the complexity of different food substrates, the diversity of target molecules, the matrix-dependent variation in the identifiable concentration observed above, and the stability and cost of sensing platforms. With further optimization, the method could be extended to other contaminant classes, such as pesticide residues and heavy metals, in a wider range of food matrices.

## 4. Conclusions

In this study, one to five layers of AgNP thin films were assembled using a liquid–liquid interface self-assembly method to construct a series of layer-tunable AgNP structures and obtain a graded set of vertically coupled gap structures. The relationship between the stacking number and the effective SERS enhancement was studied using CV as a probe molecule. The results showed that the SERS intensity increased from one to three layers and then declined, which was mainly due to the competition between the accumulation of vertically coupled hot spots and the finite optical penetration depth of the excitation laser in the densely packed metallic film. Theoretical simulation results further confirmed that the enhancement field in the gaps beyond the third AgNP layer was significantly reduced, and this prediction was directly verified by placing monolayer WS_2_ beneath the stack as a fixed-depth Raman marker. The point-to-point RSD of the CV signal at 1617 cm^−1^ over 30 positions on a single substrate was lowest for the three-layer film (5.52%), compared with 10.15% for the five-layer film; the substrate-to-substrate reproducibility was not evaluated in this work. In addition, we used the optimized three-layer AgNP system to achieve effective detection of four structurally related fluoroquinolones, namely, OFL, ENR, NOR, and CIP, with detection limits of 10^−8^ M, 10^−7^ M, 10^−7^ M, and 10^−7^ M, respectively. In actual complex systems, this method enabled the detection of antibiotic residues in spiked chicken extracts, with the lowest identifiable concentrations being 10^−7^ M for OFL, 10^−6^ M for CIP and 10^−5^ M for ENR and NOR, offering a practical screening tool for food safety monitoring. These results advance the understanding of layer-tunable multilayer gap systems and illustrate their potential in analytical applications. In the future, by matching the stacking number to the optical penetration depth at other excitation wavelengths and nanoparticle sizes, the approach could yield further improvements in sensitivity and detection limits for SERS analysis of complex samples.

## Data Availability

Data will be made available on request.

## Supplementary Materials

The following supporting information can be downloaded at https://www.mdpi.com/article/10.3390/nano16181184/s1, Figure S1: (a) UV–Vis absorption spectrum of the AgNP colloid. (b) SEM image of the synthesized AgNPs. Figure S2: (a) TEM image of the synthesized AgNPs (scale bar: 100 nm). (b) Size distribution of the AgNPs obtained by measuring 100 particles in the TEM images with Nano Measurer 1.2; The solid line is a fitted distribution curve. Figure S3: Conventional Raman spectrum of 0.1 M CV solution on a bare silicon wafer, recorded in the liquid state without a SERS substrate. Figure S4: Intensity of the CV band at 1617 cm^−1^ measured at 30 randomly selected positions on the (a) one-layer; (b) two-layer; (c) three-layer; (d) four-layer; and (e) five-layer AgNP films (10^−7^ M CV; 633 nm excitation; 1 s acquisition). The ordinate of each panel starts at zero at the lower frame, the vertical bar indicates the intensity scale, the red dashed line marks the mean of the 30 values, and the RSD is given in each panel. Figure S5: Three-layer AgNP films SERS spectra for different concentrations of CV (10^−6^–10^−10^ M). Figure S6: SERS detection spectrum of blank Three-layer AgNP films substrate: 30 spectra recorded at randomly selected positions. Figure S7: SERS spectra of CV with 10^−10^ M on the three-layer AgNP film. Figure S8: Raman spectra of WS_2_ on silicon at 633 nm; the dashed lines mark the E_2g_ and A_1g_ bands. Figure S9: Solid-state Raman spectra and molecular structures of four fluoroquinolone antibiotics. (a) OFL; (b) ENR; (c) NOR; (d) CIP. The spectra were recorded from the powders (633 nm excitation; 10 s acquisition). Each spectrum is the average of two acquisitions. Figure S10: Peak intensity as a function of the logarithmic concentration for the four fluoroquinolone antibiotics. (a) OFL at 1409 cm^−1^; (b) ENR at 1386 cm^−1^; (c) NOR at 1384 cm^−1^; (d) CIP at 1378 cm^−1^. Figure S11: SERS spectra of ethyl acetate and blank chicken extract obtained using the optimized three-layer AgNP film. Table S1: Main Raman bands of the four fluoroquinolones in the solid state (Figure S9) and in the SERS spectra recorded on the three-layer AgNP film (Figure 5), with tentative band-by-band assignments.

## Abbreviations

The following abbreviations are used in this manuscript:

AgNP_n_	AgNP film stack of n layers
AgNPs	silver nanoparticles
CIP	ciprofloxacin
CV	crystal violet
ENR	enrofloxacin
FIB-SEM	focused ion beam scanning electron microscopy
FQs	fluoroquinolones
HPLC	high-performance liquid chromatography
LC-MS/MS	liquid chromatography–tandem mass spectrometry
MRL	maximum residue limit
NOR	norfloxacin
OFL	ofloxacin
NL	nominal layer (number of interfacial transfer cycles)
RSD	relative standard deviation
SD	standard deviation
SEM	scanning electron microscopy
TEM	transmission electron microscopy
SERS	surface-enhanced Raman scattering
WS_2_	tungsten disulfide
WS_2_/AgNP_n_	WS_2_ buried beneath an n-layer AgNP film
