# Supplementary material for: Surface-Enhanced Raman Spectroscopy Method for Sensitive Detection of Antibiotic Residues by Layer-Optimized Multilayer Nanoparticle Film Structures
Source: Nanomaterials (Basel). 2026 Sep 19;16(18):1184. doi: 10.3390/nano16181184 (PMC13610879; doi:10.3390/nano16181184)
Supplement: Supplementary file 1 [file nanomaterials-16-01184-s001.zip › nanomaterials-4545073-supplementary.pdf]

Surface-enhanced Raman spectroscopy method for sensitive detection of antibiotic residues by layer-optimized multilayer nanoparticle film structures

Hongyan Wang [1], Jialing Wei [1], Yu Hu [1], Cong Wang [1] and Miao Qin [1],*

[1] Key Laboratory of Spin Electron and Nanomaterials of Anhui Higher Education Institutes, School of New Energy Materials and Chemical Engineering, Suzhou University, Suzhou, Anhui 234000, China

* Correspondence: mqin@mail.ustc.edu.cn; Tel.: +86 187 1211 1566

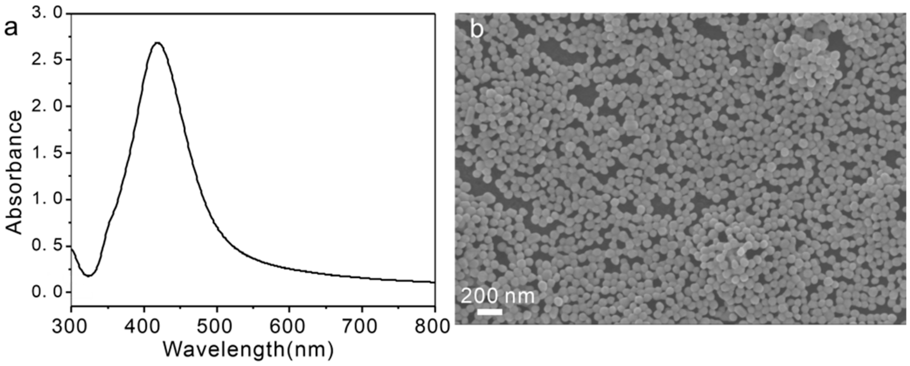

Figure S1. a. UV–Vis absorption spectrum of the AgNP colloid. b. SEM image of the synthesized AgNPs.

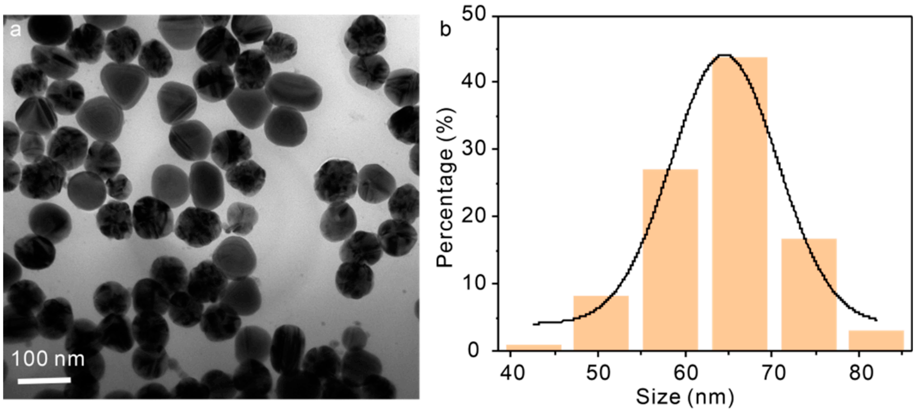

Figure S2. a. TEM image of the synthesized AgNPs (scale bar: 100 nm). b. Size distribution of the AgNPs obtained by measuring 100 particles in the TEM images with Nano Measurer 1.2; The solid line is a fitted distribution curve.

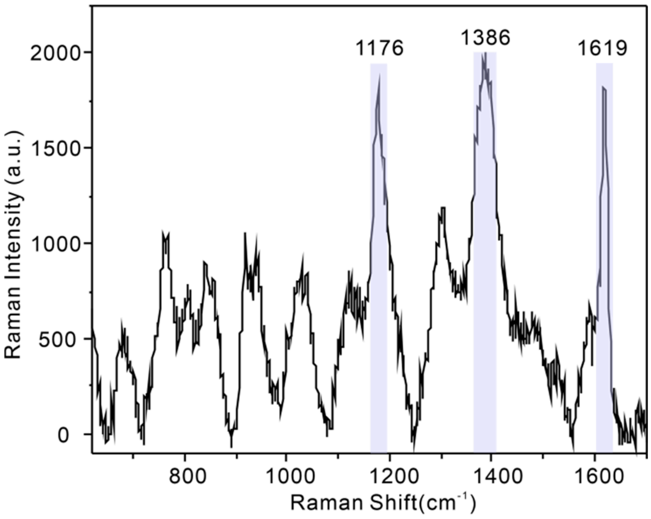

Figure S3. Conventional Raman spectrum of 0.1 M CV solution on a bare silicon wafer, recorded

in the liquid state without a SERS substrate.

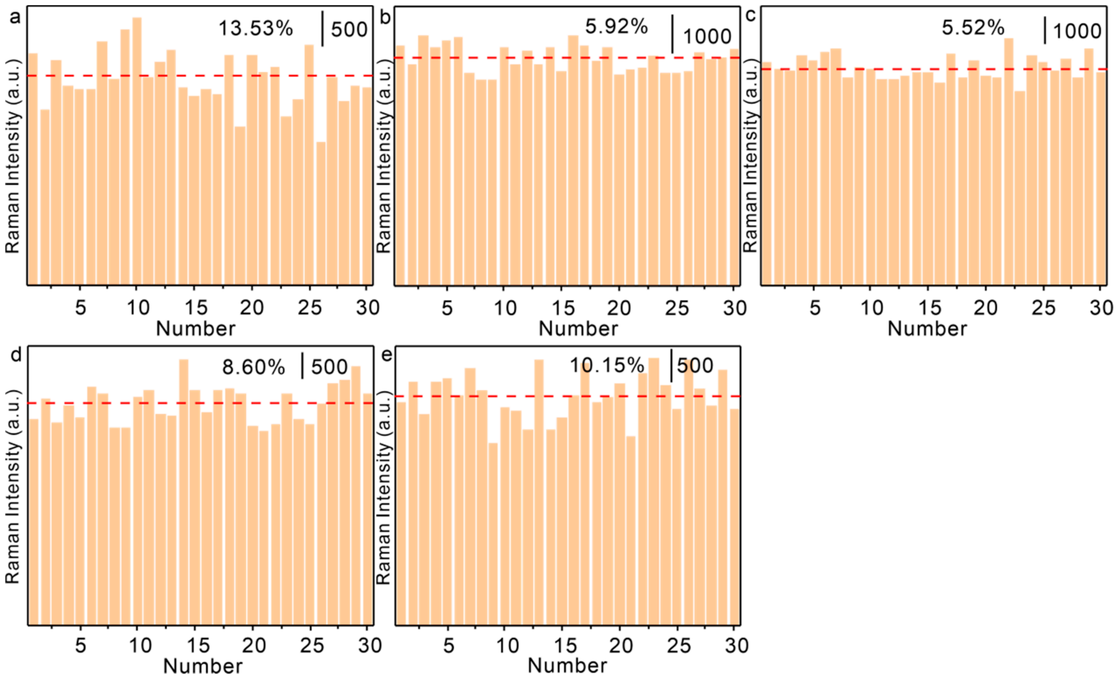

Figure S4. Intensity of the CV band at 1617 cm^{-1} measured at 30 randomly selected positions on the a. one-layer, b. two-layer, c. three-layer, d. four-layer and e. five-layer AgNP films (10^{-7} M CV; 633 nm excitation; 1 s acquisition). The ordinate of each panel starts at zero at the lower frame, the vertical bar indicates the intensity scale, the red dashed line marks the mean of the 30 values, and the RSD is given in each panel.

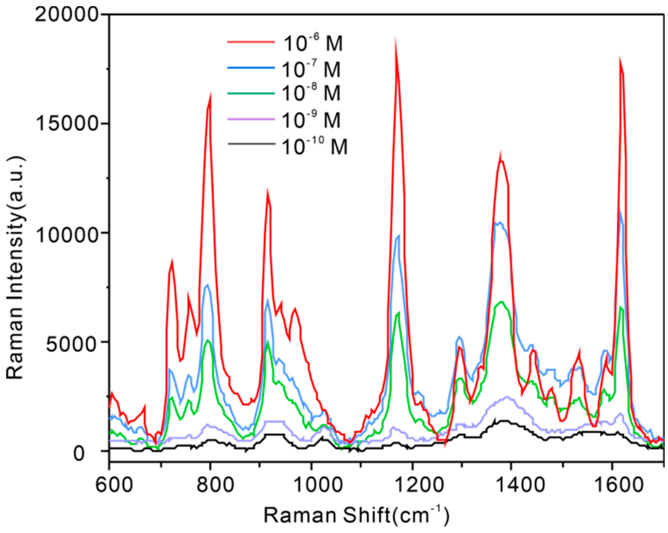

Figure S5. Three-layer AgNP films SERS spectra for different concentrations of CV (10^{-6}–10^{-10} M).

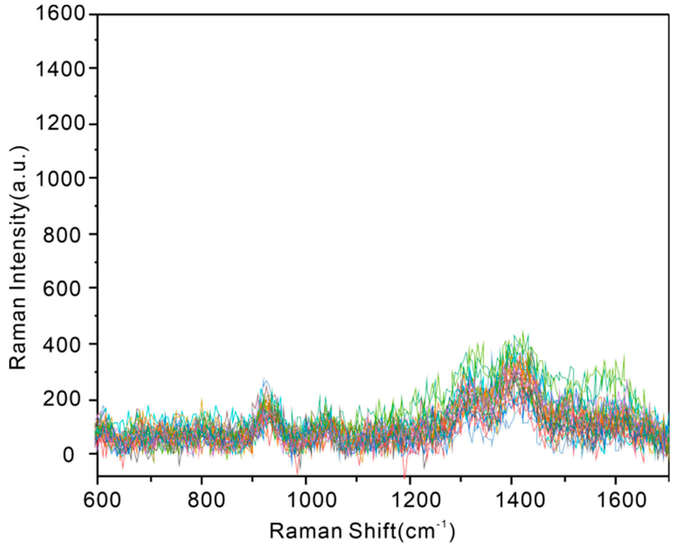

Figure S6. SERS detection spectrum of blank Three-layer AgNP films substrate: 30 spectra recorded at randomly selected positions.

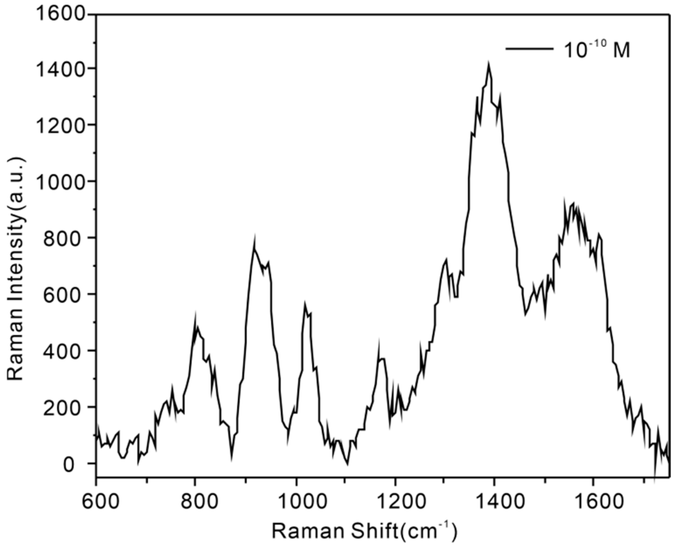

Figure S7. SERS spectra of CV with 10^{-10} M on the three-layer AgNP film.

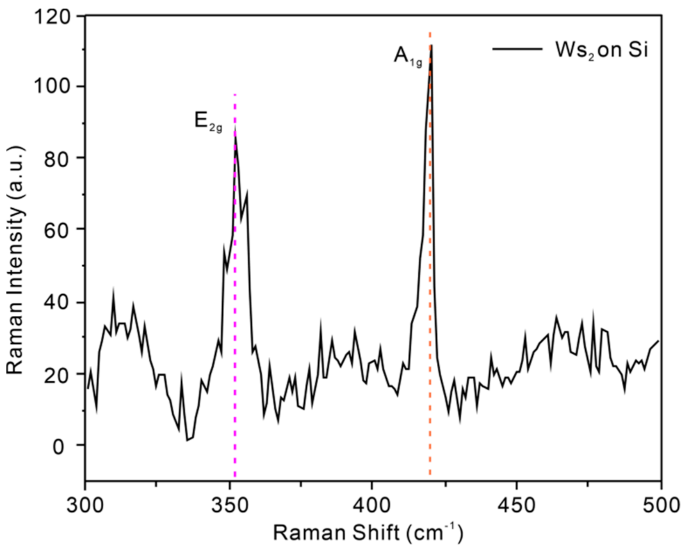

Figure S8. Raman spectra of WS2 on silicon at 633 nm; the dashed lines mark the E_{2g} and A_{1g} bands.

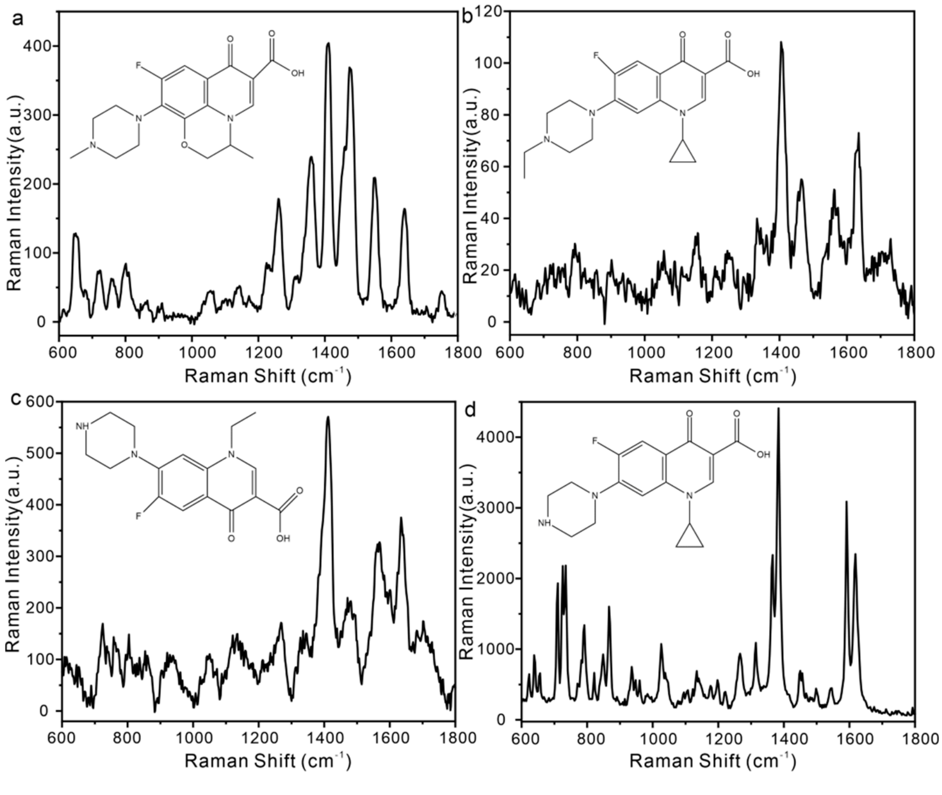

Figure S9. Solid-state Raman spectra and molecular structures of four fluoroquinolone antibiotics. a. OFL. b. ENR. c. NOR. d. CIP. The spectra were recorded from the powders (633 nm excitation; 10 s acquisition). Each spectrum is the average of two acquisitions.

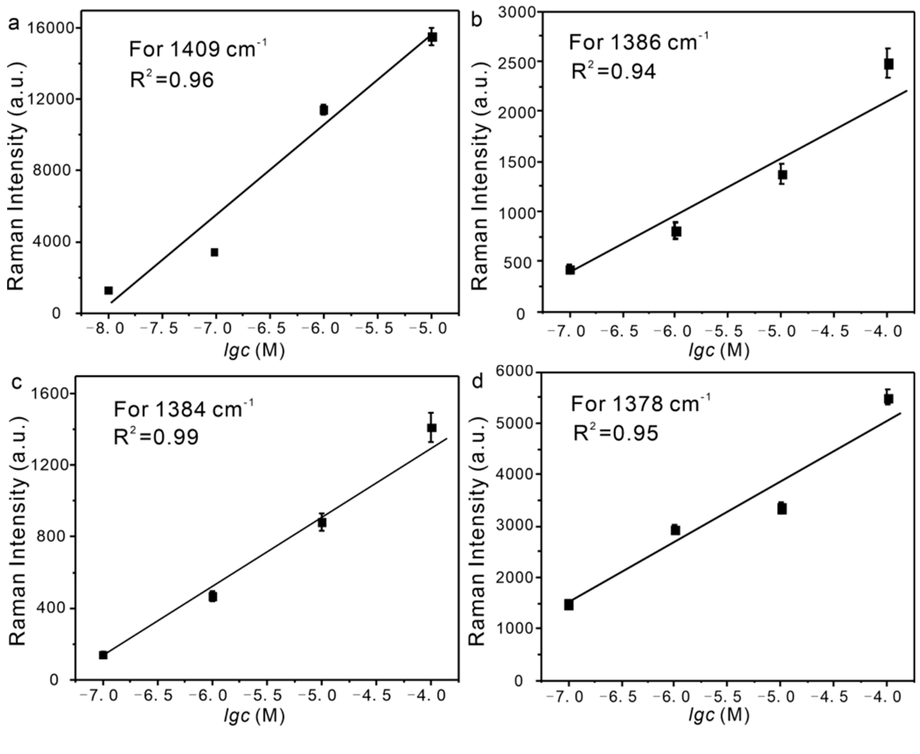

Figure S10. Peak intensity as a function of the logarithmic concentration for the four fluoroquinolone antibiotics. a. OFL at 1409 cm^{-1}. b. ENR at 1386 cm^{-1}. c. NOR at 1384 cm^{-1}. d. CIP at 1378 cm^{-1}.

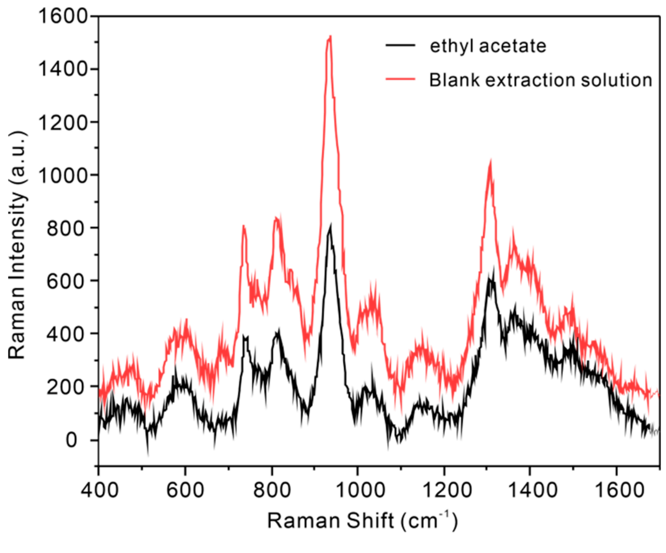

Figure S11. SERS spectra of ethyl acetate and blank chicken extract obtained using the optimized three-layer AgNP film.

Table S1. Main Raman bands of the four fluoroquinolones in the solid state (Figure S9) and in the SERS spectra recorded on the three-layer AgNP film (Figure 5), with tentative band-by-band assignments.

Compound	Solid-state band (cm^{-1})	SERS band (cm^{-1})	assignment	Ref.
OFL	757	—	N–C–C–C deformation	[1]
	1357	1357	H–C–N–C deformation	[1]
	1409	**1409**	Quinolone ring stretching; O–C–O symmetric stretching	[2,3]
	1474	—	CH_3 asymmetric bending / CH_2 bending	[3]
	—	1510	C–C stretching and out-of-plane C–H sway vibration	[4]
	1549	—	C–N and C–O stretching	[1]
ENR	1250	1256	CH_2 bending	[3]
	1341	—	—	
	—	**1386**	Quinolone ring stretching ; symmetric stretching of the carboxyl group	[1,3]
	1461	1464	CH_2 bending	[3]
	1558	1549	C=O stretching	[3]
	1632	1630	C=C stretching (aromatic ring)	[3]
NOR	1302	—	Mixed vibration	[2]
	—	**1384**	O–C–O symmetric stretching ; quinolone ring stretching	[2,3]

	1410	—	O–C–O symmetric stretching	[2]
	1623	—	C=C stretching (aromatic rings)	[3]
CIP	1265	—	In-plane ring C–H bending	[3]
	1365	—	Mixed vibration of the piperazine ring	[3]
	1384	**1378**	O–C–O stretching ; quinolone ring stretching	[3,5]

Reference

1. Kalaimagal, K.; Gunasekaran, S. FTIR, FT-Raman and Density Functional Theory Studies on Ofloxacin. *Int. J. ChemTech Res.* **2015**, *8*, 1403–1416.
2. Qin, M.; Ma, M.; Yan, J.; Wang, J.; Zhu, S.; Wang, C.; Ge, M. Rapid Detection of Fluoroquinolone Antibiotic Residues in Poultry Feathers via Liquid–Liquid Microextraction Combined with Surface-Enhanced Raman Spectroscopy. *J. Mod. Opt.* **2026**, *73*, 356–363, doi:10.1080/09500340.2025.2583990.
3. Jiang, Y.; Wang, X.; Zhao, G.; Shi, Y.; Wu, Y. In-Situ SERS Detection of Quinolone Antibiotic Residues in Aquaculture Water by Multifunctional Fe_3O_4@mTiO_2@Ag Nanoparticles. *Spectrochim. Acta, Part A* **2023**, *302*, 123056, doi:10.1016/j.saa.2023.123056.
4. Wu, Y.; Huang, J.; Shang, L.; Chen, Q.; Peng, F.; Liu, T.; Luo, Z.; Liu, M.; Zhao, J.; Huang, S. Exploring SERS Methods for Rapid Determination of Ofloxacin Residues in Egg White. *Spectrochim. Acta, Part A* **2026**, *345*, 126810, doi:10.1016/j.saa.2025.126810.
5. Li, D.; Sun, Y.; Pei, J.; Yu, X.; Tian, Z.; Xu, H. Au-SnO_2 Resonator for SERS Detection of Ciprofloxacin. *Microchem. J.* **2024**, *203*, 110830, doi:10.1016/j.microc.2024.110830.
